# Effect of Voluntary Wheel Running on Striatal Dopamine Level and Neurocognitive Behaviors after Molar Loss in Rats

**DOI:** 10.1155/2017/6137071

**Published:** 2017-12-05

**Authors:** Linlin Zhang, Yi Feng, Wenliang Ji, Jianzhang Liu, Kun Liu

**Affiliations:** ^1^Capital University of Physical Education and Sports, Beijing 100191, China; ^2^Beijing Xicheng District Desheng Community Health Service Center, Beijing 100120, China; ^3^Peking University School and Hospital of Stomatology, Beijing 100081, China; ^4^Department of Psychiatry, Yale University School of Medicine, New Haven, CT 06511, USA

## Abstract

The aim of the present study is to evaluate the effect of voluntary wheel running on striatal dopamine level and behavior of cognition and emotion in molar loss rats. Twenty-four Sprague-Dawley rats were enrolled in this study and randomly divided into following 4 groups: control group (C group), molar loss group (ML group), 1-week physical exercise before molar loss group (1W-ML group), and 4-week physical exercise before molar loss group (4W-ML group). The rats both in 4W-ML and 1W-ML groups were placed in the voluntary running wheel in order to exercise for 4 weeks and 1 week, respectively. Then, the rats in 4W-ML, 1W-M, and ML groups received bilateral molar loss operation. After 10 days, striatal dopamine level was detected by in vivo microdialysis coupled with high-performance liquid chromatography (HPLC) and electrochemical detection. All the rats received behavior test after microdialysis detection. The behavior tests including passive avoidance test were used to assess cognition and elevated plus maze test for emotion. The results indicated that voluntary wheel running promoted striatal dopamine level in rats of molar loss. Behavioral data indicated that voluntary wheel running promoted cognition and emotion recovery after molar loss. Therefore, we concluded physical exercise significantly improved the neurocognitive behaviors and increased the striatal dopamine level after molar loss in rats.

## 1. Introduction

It is well established that tooth loss is a known risk factor of Alzheimer's disease (AD) [1]. As a common disorder in senior population, loss of teeth could adversely affect human cognition and emotion [2, 3]. Clinical medicine has established that tooth loss in patients can induce neuronal cell loss and memory impairment [4, 5]. Animal research also demonstrated that molar loss can cause the functional deterioration of cognition [6]. Previous studies have demonstrated that the rat with molar loss broke the balance of the cholinergic neuronal system and caused the impairment of cognition [7]. In addition, a preliminary quantitative study revealed that wide range of emotional effects was caused by tooth loss, such as loss of self-confidence and anxiety [8]. Further studies have shown that physical exercise, aerobic fitness exercise in particularly, had a positive effect on multiple aspects of brain function [9–11]. Neurochemistry studies indicated that physical exercise increased the antioxidant ability and glucose level to enhance cognition and emotion [12–14]. Neurobiology research also confirmed that aerobic exercise predominately employed the action of BDNF and the new growth of synaptic plasticity [15–17]. In addition, dopamine acts as a classic neurotransmitter in the brain, which plays an important role in cognitive and emotive aspects [18–20]. Numerous studies have shown that dopamine degeneration caused significant alteration in cognitive and emotive function by medicating striatal dopamine pathways [21-22]. Striatum is one of the four major dopamine pathways in the brain, partially involved in reward and in the reinforcement of memory consolidation. Degeneration of dopamine-producing neurons in striatum complex leads to diminished concentrations of dopamine in the nigrostriatal pathway, leading to reduce function and the characteristic symptoms, such as motor ability and cognitive impairment [23–25]. Therefore, this study was designed to explore the effect of voluntary wheel running on striatal dopamine level and neurocognitive behaviors after molar loss in rats. These findings may provide a theoretical basis for the clinical prevention of Alzheimer's disease.

## 2. Materials and Methods

### 2.1. Animals

Adult male Sprague-Dawley rats (3 months of age, weighting 300 ± 50 g at the time of surgery) were obtained from Experimental Animal Center of Peking University. Rats (*n* = 24) were enrolled in this study and randomly divided into following 4 groups: control group (C group, *n* = 6), molar loss group (ML group, *n* = 6), 1-week physical exercise before molar loss group (1W-ML group, *n* = 6), and 4-week physical exercise before molar loss group (4W-ML group, *n* = 6). The rats in both 4W-ML and 1W-ML groups were placed in the voluntary running wheel in order to exercise for 4 weeks or 1 week. Then, the rats in 4W-ML, 1W-ML, and ML groups received bilateral molar loss operation. In the C group, bilateral maxillary molar teeth remained intact. All rats were housed under 12 h light/dark cycle and had a sufficient amount of food and water.

### 2.2. Voluntary Wheel Running

The rats in both 4W-ML and 1W-ML groups received voluntary wheel running. Two rat groups were given free access to running wheels (wheel circumference, 100 cm; Harvard Apparatus) in their cages for 4 weeks or 1 week. A magnetic counter was installed to the running wheel in order to record wheel revolutions [26]. The distance was obtained by wheel revolutions multiplied by the circumference of the wheel. Those rats that cannot adapt new circumstances will be removed.

### 2.3. Molar Loss Surgery

Bilateral maxillary molar teeth were extracted from rats in the 4W-ML, 1W-ML, and ML groups after being anesthetized with chloral hydrate (350 mg/kg, i.p.). The rats in the C group were anesthetized, but no teeth were extracted. To provide a suitable recovery time after teeth extraction, experimental dentures were fitted to rats. Experimental dentures were produced from an impression made of silicone impression material and a resin tray. Occlusal adjustments were made until maxillomandibular incisor contacts were obtained [27].

### 2.4. In Vivo Microdialysis and High-Performance Liquid Chromatography

In vivo microdialysis was performed by implanting microdialysis probe into the striatum through the guide cannula by continuously perfusing with artificial cerebrospinal fluid (126 mM NaCl, 2.4 mM KCl, 1.1 mM CaCl_2_, 0.85 mM MgCl_2_, 27.5 mM NaHCO_3_, 0.5 mM Na_2_SO_4_, and 0.5 mM KH_2_PO_4_, pH = 7.0) at a flow rate of 2 *μ*L/min driven by a microinjection pump (CMA/100, CMA Microdialysis AB, Stockholm, Sweden). After 90 min, microdialysate in the striatum were collected for 60 minutes; each sample was collected in a 250 *μ*L tubes for 10 min for a total of 6 tubes. Each tube was placed in an ice box containing 15 L of 10 mmol HCL. Then, dopamine level in the samples was measured using HPLC with electrochemical detection. The flow rate of the mobile phase (50 mM NaH_2_PO_3_, 2 mM decanesulfonic acid, 0.7 mM ethylenediaminetetraacetic acid, 11% *v*/*v* acetonitrile, and 11% *v*/*v* methanol, pH = 6.0) was 1 *μ*L/min.

### 2.5. Passive Avoidance Test

Cognitive function was assessed by passive avoidance test [28]. After the learning trail, the retention test was measured 24 h after the learning trial. Each animal was put in an illuminated compartment, and the door opened after 2 minutes. Latency to enter the dark compartment was recorded to a maximum of 300 s. Animals that did not enter the dark chamber during the retention test were allotted a latency of 300 s [29].

### 2.6. Elevated Plus Maze Test

Emotion was assessed by elevated plus maze test [30]. The elevated plus maze was made of plastic and consisted of two white open arms (25 × 8 cm), two black enclosed arms (25 × 8 × 20 cm), and a central platform (8 × 8 × 8 cm) in the form of a cross. The maze was placed 50 cm above the floor. Rats were individually placed in the center with their heads directed toward one of the closed arms. The total time spent in each arm or in the center and the total number of entries into each arm were analyzed by video monitoring for 5 min. After 5 min, rats were removed from the maze and returned to their home cage. The maze was then cleaned with a solution of 70% ethyl alcohol and permitted to dry between tests [31].

### 2.7. Statistical Analysis

Data were analyzed by GraphPad Prism 5. A two-way analysis of variance (ANOVA) was used to analyze the data. The comparison of cognition and emotion was made with two-tailed Student's *t* test.

## 3. Results

### 3.1. Dynamic Changes of Striatal Dopamine Level in Each Group

**Figure 1:**
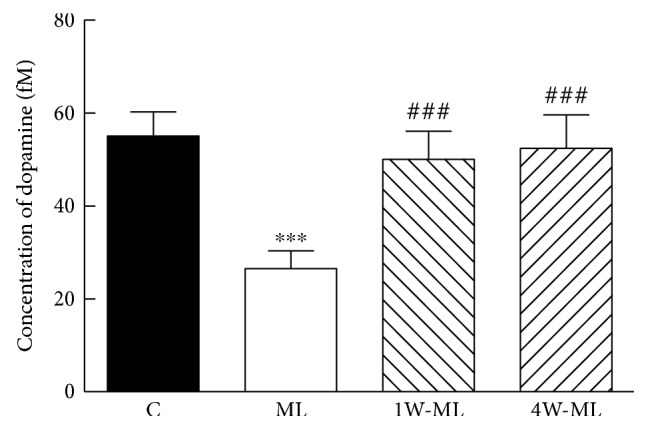
Dynamic changes of striatal dopamine level in each group. ^∗∗∗^*P* < 0.01 compared with C group. ^###^*P* < 0.01 compared with ML group.

Figure 1 shows dynamic changes of striatal dopamine level in each group. The dopamine level in C, ML, 1W-ML, and 4W-ML groups was 55.02 ± 9.05 fmol, 26.48 ± 6.67 fmol, 50.01 ± 10.52 fmol, 52.34 ± 12.54 fmol, respectively. Compared with C group, the extracellular dopamine level of ML group significantly decreased (*P* < 0.001). Compared with ML group, the extracellular dopamine level of 1W-ML and 4W-ML group significantly increased (*P* < 0.001). However, the extracellular dopamine level has no significant difference between 1W-ML and 4W-ML groups (*P* > 0.05). The results indicated that 1-week or 4-week volunteer wheel running increased striatum dopamine level after molar loss.

### 3.2. Passive Avoidance Test

**Figure 2:**
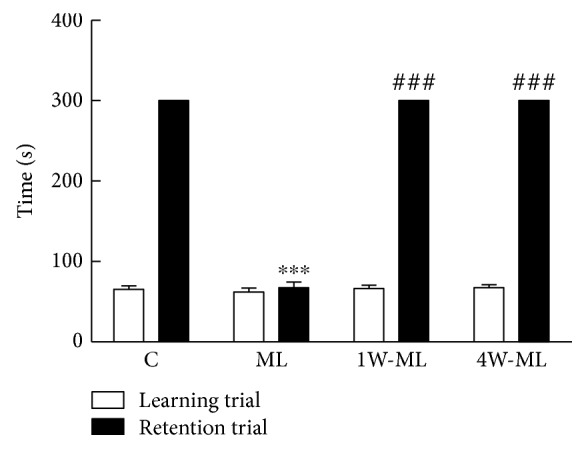
The latency time of rats in learning trial and retention trial. ^∗∗∗^*P* < 0.001 compared with C group. ^###^*P* < 0.01 compared with ML group.

Figure 2 shows that the latency time of passive avoidance test in each group. In learning trial, the time in C, ML, 1W-ML, and 4W-ML group was 65.5 ± 11.89 s, 62 ± 13.41 s, 66.5 ± 8.56 s, and 67.5 ± 10.27 s and 300 s, 67.5 ± 16.69 s, 300 s, and 300 s in retention trial. There was no significant difference in the learning trial for each group (*P* > 0.05). Compared with C group, the retention time of ML group was significantly shorter (*P* < 0.001). Compared with ML group, the retention time of 1W-ML and 4W-ML groups was significantly longer (*P* < 0.001). However, the retention time has no significant difference between 1W-ML and 4W-ML groups (*P* > 0.05). The results indicated that 1-week and 4-week volunteer wheel running can promote cognition after molar loss.

### 3.3. Elevated Plus Maze Test

**Figure 3:**
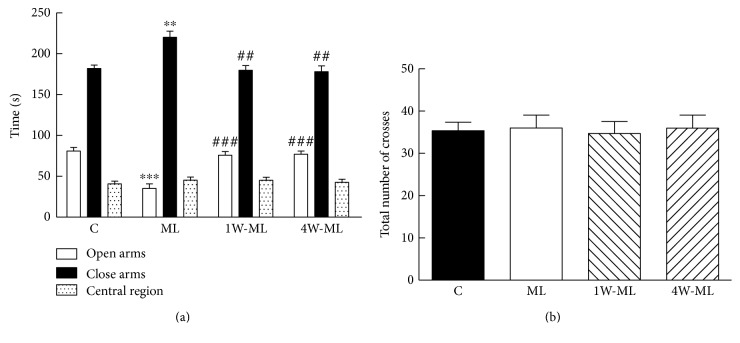
(a) Time spent of four rat groups in each arm. ^∗∗∗^*P* < 0.001 and ^∗∗^*P* < 0.01 compared with C group. ^###^*P* < 0.001 and ^##^*P* < 0.01 compared with ML group. (b) Total number of crosses in each group.

Figure 3(a) shows that the time spent of each rat groups in each arm. Compared with C group, the time spent of open arms in the ML group was significantly shorter (*P* < 0.001), adversely close arms (*P* < 0.01). Compared with ML group, the time spent of open arms in the 1W-ML and 4W-ML groups was significantly longer (*P* < 0.001), adversely close arms (*P* < 0.01). However, the time spent in each arm has no difference between 1W-ML and 4W-ML groups (*P* > 0.05). Meanwhile, there was no significant difference in the central region time for each group (*P* > 0.05).  Figure 3(b) shows that total number of crosses in three groups. But there was no significant difference in the total number of crosses for each group (*P* > 0.05). The results indicated that 1-week or 4-week volunteer wheel running can promote emotion after molar loss.

## 4. Discussion

Alzheimer's disease (AD) can be considered as the most common cause of cognitive dysfunction among the aged, and tooth loss might be a risk factor for Alzheimer-type dementia [32–34]. New advances have shown that tooth loss caused the decline of cognition [35–37]. Previous research used passive avoid test to evaluate cognition, which shows that molar loss may cause accumulation of the amyloid cascade in the brain that leads to cognitive impairment [38]. Thus, we chose passive avoidance test to assess cognition in rats. We also choose elevated plus maze test to evaluate emotion. The results show that the cognition and emotion were worse after molar loss. Therefore, we successfully established the rat model of cognitive and emotive dysfunction after molar loss in this experiment. Previous study has shown that molar loss-induced cognitive and emotive impairments are related to neural cell loss [38-39]. In this study, we also demonstrated that molar loss-induced cognitive and emotive impairments are associated with the decrease of striatal dopamine level. Clinical studies have demonstrated that cognitive impairment had pathophysiology of dopamine system by the loss of midbrain neurons that synthesize the neurotransmitter dopamine [40-41]. Animal study also demonstrated that depression and anxiety might be associated with a specific loss of dopamine innervation in the limbic system [42]. Thus, it is reasonable to suggest that the decrease of striatal dopamine level can be one of the important reason of cognitive and emotive impairment after molar loss.

However, therapeutic strategies for the cognitive and emotive impairment after molar loss have not been well established so far. Molecular chemistry and biology highlight the promoted effect of physical exercise on cognition and emotion [43–45]. Some research has shown that exercise increased synaptic plasticity and the level of BGF and promoted cognition [46-47]. Other research also have shown that exercise can enhance cell proliferation, thereby alleviating anxiety-like behavior and improve emotion [48-49]. Especially, studies also indicated that physical exercise, voluntary wheel running in particularly, can be one of the best ways to promote cognition and emotion [50-51]. So, the present study chose voluntary wheel running as intervention approach. Our behavioral data indicated that voluntary wheel running promoted cognitive and emotive recovery after molar loss. As reported previously, physical exercise promoted the new growth of synaptic plasticity and the level of brain glucose and improve cognition and emotion thereafter [52–54]. In our research, we demonstrate that 4-week and 1-week voluntary wheel running promoted cognitive and emotive recovery after molar loss is associated with the increase of striatal dopamine level. Previous studies have demonstrated that physical exercise increased antioxidant defense and dopamine levels [55–57]. The increased dopamine level is related to recovery of cognitive and emotive impairments. Thus, we suggest that the increased of striatal dopamine level can be one of the reasons to induce that voluntary wheel running promotes cognition and emotion after molar loss in rats.

It is worth to mention that there was no difference between 4 weeks and 1 week of physical exercise-promoted cognition and emotion after molar loss in dopamine concentration aspect. Since previous research have found that small amount of physical exercise could be beneficial to cognitive function, it is reasonable that 1 week of physical exercise is enough to promote cognition and emotion after molar loss in dopamine concentration aspect. Although we did not find difference of dopamine level in striatum after 1 week and 4 weeks of physical exercise in our experiment condition, it is still possible that dopamine dynamic change in different brain regions could be induced by different physical exercise processes. In addition, whether there is difference in other aspects, such as other cognitive behavioral models or cellular levels, needs to be studied further.

## 5. Conclusions

Physical exercise significantly promoted the cognition and emotion in rats of molar loss by increasing the striatal dopamine level. However, it is necessary to further study other neurochemicals related to dopaminergic system to elucidate more underlying mechanisms of physical exercise-promoted cognition and emotion after molar loss.
